# Reduce Muscle Fibrosis through Exercise via NRG1/ErbB2 Modification in Diabetic Rats

**DOI:** 10.1155/2020/6053161

**Published:** 2020-05-13

**Authors:** Majid Amani, Masoud Rahmati, Mohammad Fathi, Hasan Ahmadvand

**Affiliations:** ^1^Department of Physical Education and Sport Sciences, Faculty of Literature and Human Sciences, Lorestan University, Khoramabad, Iran; ^2^Department of Biochemistry, Faculty of Medicine, Lorestan University of Medical Sciences, Khorramabad, Iran

## Abstract

Diabetic myopathy refers to the manifestations in the skeletal muscle as a result of altered glucose homeostasis which reflects as fibrosis. Since physical exercise has been indicated a protective strategy for improving glucose metabolism in skeletal muscle, we tested a hypothesis under which the endurance exercise training could reverse the produced skeletal muscle fibrosis by diabetes. Eight-week-old male Wistar rats were randomly assigned into four groups including healthy control (HC), healthy trained (HT), diabetic control (DC), and diabetic trained (DT) groups. Diabetes was induced by a single intraperitoneal injection of streptozotocin (STZ; 45 mg/kg). Rats in the HT and DT groups carried out an exercise program on a motorized treadmill for five days a week over six weeks. Skeletal muscle levels of NRG1and ErbB2 were measured by the Western blot method. Exercise training decreased blood glucose levels in the DT group. Induction of diabetes increased skeletal muscle fibrosis in both the fast extensor digitorum longus (EDL) and slow soleus muscles, while endurance training modified it in diabetic trained rats. Moreover, muscle NRG1and ErbB2 levels were increased in diabetic rats, while training modified muscle NRG1and ErbB2 levels in diabetic trained rats. Our study provides novel evidence that endurance training could modify skeletal muscle fibrosis through NRG1/ErbB2 modification in STZ-induced diabetic rats.

## 1. Introduction

According to the International Diabetes Federation (IDF), there were approximately 425 million people living with diabetes worldwide in 2017, out of which 5% to 10% are estimated to have T1DM (42.5 to 95 million) [1]. Skeletal muscle responds to many stimuli through changes in muscle size, distribution of fiber types, or metabolism [2]. Diseases like diabetes can affect the health of skeletal muscle and affect their function. Weight loss, muscle atrophy, and increased blood glucose can affect the muscle health in the long term [3]. Diabetic myopathy, which is characterized by decreased muscle capacity, strength, and mass [4], is a complication of diabetes mellitus and directly affects the mortality rate due to the fact that the skeletal muscle is the largest source of glucose consumption, and thus, any changes in its health can affect the metabolism of whole-body glucose [5]. GLUT4 is an important component in the transfer of glucose into the muscle cell. It can enter the cell surface through exercise and cause glucose to enter the cell (independent of insulin) and neuregulin 1 (NRG1) can facilitate the above process [6].

NRG1 is a growth factor from the family of epithelial growth factors and acts as the autocrine and paracrine method through receptor tyrosine kinases (RTKs) including ErbB2, ErbB3, and ErbB4. NRG1 plays important physiological roles in the central nervous system, liver, and skeletal muscle [7–10]. NRG1 plays an important role in improving the glucose tolerance in the liver and soleus muscles in young and old rats [11]. The evidence suggests that the ErbB family can play a central role in the development of many complications of diabetes [12]. NRG signals are facilitated by activating the receptor tyrosine kinases or ErbBs. ErbB2 is activated by NRG1 adhesion and leads to the phosphorylation of cytoplasmic tyrosine residues that begin various downstream signal events [13]. In addition, it seems that NRG1 regulates the metabolism, so that the stimulation of NRG1 of the skeletal muscle facilitates the glucose uptake by increasing insulin effects; and evidence suggests that contractile activities in in vivo conditions activate NRG1/ErbB2 [8]. These studies indicate that glucose metabolism disorder in diabetes may lead to skeletal muscle dysfunction in the NRG1/ErbB2 pathway, thereby affecting functions of the skeletal muscle.

Hyperglycemia, which is a complication of diabetes, can affect the extracellular matrix (ECM) regeneration through metabolic and hemodynamic pathways [14]. In pathological conditions such as diabetes, the ECM production becomes higher than its destruction and causes fibrosis [15]. Inappropriate rearrangement of diabetic muscle ECM, which is characterized by the higher collagen presence, reduces the total amount of muscle tissue and inevitably affects the muscle motion and its reconstruction; and the defects affect the aging of skeletal muscles [16]. Antifibrotic effects of NRG1 have been reported in rats with diabetic cardiomyopathy [17] and cardiac, pulmonary, and skin fibrosis [9]. On the other hand, the higher expression of endogenous NRG1 in heart muscles at early stages of cardiac fibrosis expresses the counterregulatory effects of NRG1/ErbB system [9], but this feature is not known in diabetic skeletal muscles. Some studies indicate that the activation of NRG1/ErbB2 can be an effective molecular strategy in the repair and regeneration of cardiac muscle after a heart attack [18]. The exercise training is a way to activate NRG1/ErbB2 that has been able to improve the recovery and regeneration of cardiac muscle and reduce fibrosis in rats with heart attack [19]. However, the effectiveness of exercise training is unclear on muscle fibrosis caused by diabetes.

Some studies have reported the potential of NRG1 therapy in diabetes as it may suggest a new pathway in the applied treatment of diabetic myopathy; and strategies such as exercise may reduce or delay muscle fibrosis. Therefore, according to the role of NRG1/ErbB2 in the hemostasis of glucose and reduction of fibrosis, it may be effective in maintaining the structures and functions of skeletal muscles in diabetic rats. On the other hand, the aerobic exercise may reduce complications of diabetes by effecting hyperglycemia. We investigated the above hypotheses in STZ diabetic rats.

## 2. Materials and Methods

In the present study, 40 male Wistar rats weighing 200-250 g were purchased as research samples from the Animal Care Center of Lorestan University of Medical Sciences. The rats were kept in a 12- to 12-hour light-dark cycle at 21°C, and rats were given *ad libitum* access to food and water. Research protocol and all conditions of care and exercise were approved by the Ethics Committee of Lorestan University with a code of LU.ECRA.2017.12, and they could be conducted in accordance with rules governing live animal testing in Iran and the Asian Convention for protecting animals that are used in laboratory experiments and other scientific purposes. After 12 hours of food deprivation, diabetes was induced with single-phase and intraperitoneal injection of STZ solution (Sigma, St. Louis, MO; 45 mg/kg) that was solved in 0.5 mol/l fresh citrate buffer, pH 4.5 at 4°C. The nondiabetic rats received the same extent of injected citrate buffer [20]. Diabetic status of rats continued by weekly measurement of the blood glucose (without fasting and through rats' tail veins) using a glucometer (Roche Diagnostics K.K., Tokyo, Japan) until the eighth week after the STZ injection; and rats with blood glucose levels of above 350 mg/dl were included in the study. Body weight and general health of rats were monitored over a period of 8 weeks, and all animals behaved appropriately during this period. Two weeks after injection of diabetes, the rats were randomly divided into four 10-rat groups, namely, the diabetic control (DC), diabetic trained (DT), healthy control (HC), and healthy trained (HT) groups.

### 2.1. Training Protocol

The protocol of rat familiarization with run on treadmill was done over a period of 5 days, so that rats ran once a day for 10 minutes per session at a speed of 10 m/min and a zero slope on the treadmill. In the present study, the endurance training was done by moderate intense training based on a study by Rahmati et al. [20], so that the sports groups ran 5 sessions per week for 6 weeks on the treadmill with a zero-degree slope. The velocity and duration of treadmill (zero slope) gradually increased from 10 m/min to 10 minutes in the first week, 10 m/min to 20 minutes in the second week, 15 m/min to 20 minutes in the third week, 15 m/min to 30 minutes in the fourth week, and 17-18 m/min to 30 minutes in the fifth week. In order to achieve the uniform consistency, all training variables were kept constant during the final week (week 6). The training protocol consisted of two to three minutes of warming and three minutes of cooling. Most animals ran the duration of training without any encouragement, and no training shock was used during the endurance training program. All training sessions were held at the end of animals' sleep cycles between 16:00 and 18:00 [21]. In training groups, animals did training sessions without any electrical shock. During training sessions, animals did not show any signs of excessive exhaustion because boring training in diabetic animals might increase complications of diabetes by altering lactic acid levels [20].

### 2.2. Preparation of Tissues

The muscle tissue isolation was done after blood sampling. Soleus muscles and their EDL were extracted after creating a cut on the skin of animals' legs. After isolation, some specimens were immediately placed in formalin 10% for fixation to be histological studies, and the remaining specimens were placed in liquid nitrogen for analysis of proteins and then stored at -80°C for further analysis.

### 2.3. Masson's Trichrome Staining and Collagen Level Measurement

After removal of tissue samples from formalin, they were placed in 70% ethanol, 80% ethanol, 90% ethanol twice, and 100% ethanol twice each for 60 minutes. The tissues were then cleared in xylene and molded by paraffin. Tissue cuts with a thickness of 5 microns were prepared by a SLEE Cut 4060 rotary microtome device. For hydration of cuts, they were placed in 100% to 50% ethanol solutions for a minute. The sections were stuck onto slides with albumin glue, and then, the Masson's trichrome staining was carried out. The staining, which was used to detect collagen increase and measure the severity of fibrosis in muscle tissue, was performed as follows.

Cross sections were stained in Weigert's hematoxylin for 15 min, washed in distilled water, then washed in running tap water for 5 min. Muscle samples were then stained with 1% Biebrich scarlet-acid fuschin for 15 min, then washed in distilled water for 5 min. After differentiation in 2.5% phosphomolybdic-phosphotungstic acid solution for 15 min, sections were transferred directly into 2.5% aniline blue solution for 12 min. Muscle samples were then differentiated in 1% acetic acid solution for 3 min, dehydrated in 95% and 100% ethanol, then cleared in xylene. In this technique, muscle fibers stain a bright red and collagen fibers blue. Images were captured with an Olympus microscope (CX31, Japan), and in order to determine the amount of collagen present in tissue images, we used ImageJ analysis program (NIH, Bethesda, MD, USA). Serial sections in 5 rats/group were analyzed for percentage connective tissue area [22, 23].

### 2.4. Western Blot Analysis

The frozen muscle tissues were powdered in liquid nitrogen. Muscle tissues (50 mg) were homogenized in 400 *μ*l lysis buffer (20 mM Hepes, 350 mM NaCl, 20% (*v*/*v*) glycerol, 1% (*v*/*v*) Nonidet P-40, 1 mM MgCl_2_, 0.5 mM EDTA, and 0.1 mM EGTA, pH 7.9) supplemented with a protease inhibitor cocktail (Sigma-Aldrich Co.). Homogenates were then centrifuged at 12,000 g for 15 min at 4°C, and the supernatant stored at -80°C for further analysis. Protein content was measured based on the Bradford method using bovine serum albumin (BSA) as the standard (Sigma-Aldrich Co.). Then, equal quantities of proteins were separated on SDS page. The separated proteins were transferred to a PVDF membrane by a transfer apparatus at 0.35 A for 2.5 hours. The membranes were blocked with 5% dried skim milk in TBST (20 mM Tris, 150 mM NaCl, and 0.05% Tween-20). After 2 h at room temperature, the membranes were washed and incubated with primary antibody against ErbB2 (1 : 1500; ab214275, Abcam, Cambridge, MA), NRG1 (1 : 1500; ab180808, Abcam, Cambridge, MA), and GAPDH (1 : 1000; Santa Cruz Biotechnology Inc., Dallas, TX, USA). After incubating with an anti-mouse horse-radish peroxidase-conjugated secondary antibody, protein was visualized using an enhanced chemiluminescence system. The images were captured by the charge-coupled device camera (Fujifilm, Tokyo, Japan), and densitometric analysis of digitized images was quantified using TotalLab (Nonlinear Dynamics Ltd., Newcastle, UK) which were normalized using GAPDH. Five rats in each group were used for this analysis. Moreover, analysis was performed in triplicate.

### 2.5. Statistical Method

Shapiro-Wilk Test verified the normal data distribution and Levene's test examined the equality of variances. The two-way ANOVA was used to compare groups in studied variables; and Tukey's post hoc test was utilized to complete the supplementary test. Data was presented based on the mean ± SD, and the significance level was *P* ≤ 0.05. Data analysis was also done using SPSS 22.

## 3. Results

### 3.1. Changes in Blood Glucose and Body Mass

**Figure 1:**
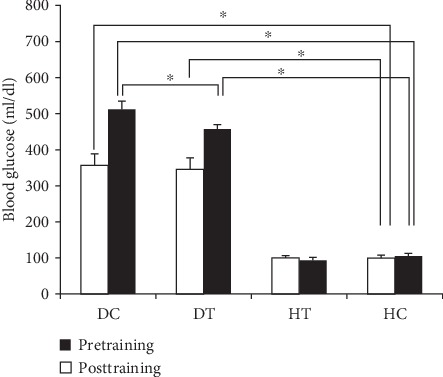
Changes of blood glucose after streptozotocin (STZ) injection (pretraining) and after 6-week exercise treatment (posttraining). The diabetic and trained group (DT) performed 6 weeks of endurance training. Data is shown as the mean ± SEM. ^∗^*P* < 0.05 vs. control rats (two-way ANOVA). DC: diabetic control rats; DT: diabetic and trained; HC: health and control; HT: health and trained. Ten rats in each group were utilized for this analysis.

STZ diabetic rats showed higher blood glucose levels (511.85 ± 4.05 vs. 105.14 ± 2.61 mg/dl) and weight loss weight loss (234 ± 15.05 vs. 198.37 ± 20.72 g). Before starting the training protocol, the blood glucose level of the DC group had a significant difference from HC (*P* = 0.001), and the significant difference was maintained at the end of six weeks. The six-week endurance training could significantly decrease the blood glucose of the DT group than the DC group (*P* = 0.002) indicating a positive effect of training on the blood glucose (Figure 1).

At the end of six weeks, the body mass of diabetic rats of the DC group significantly decreased compared to that of the HC group (*P* = 0.001), and six weeks of endurance training did not prevent this decline. Despite the lower mean weight of the HT group than the HC group (247.77 ± 9.39 vs. 258.5 ± 8.33 g), the difference was not statistically significant (Table 1).

### 3.2. Histopathological Changes

**Figure 2:**
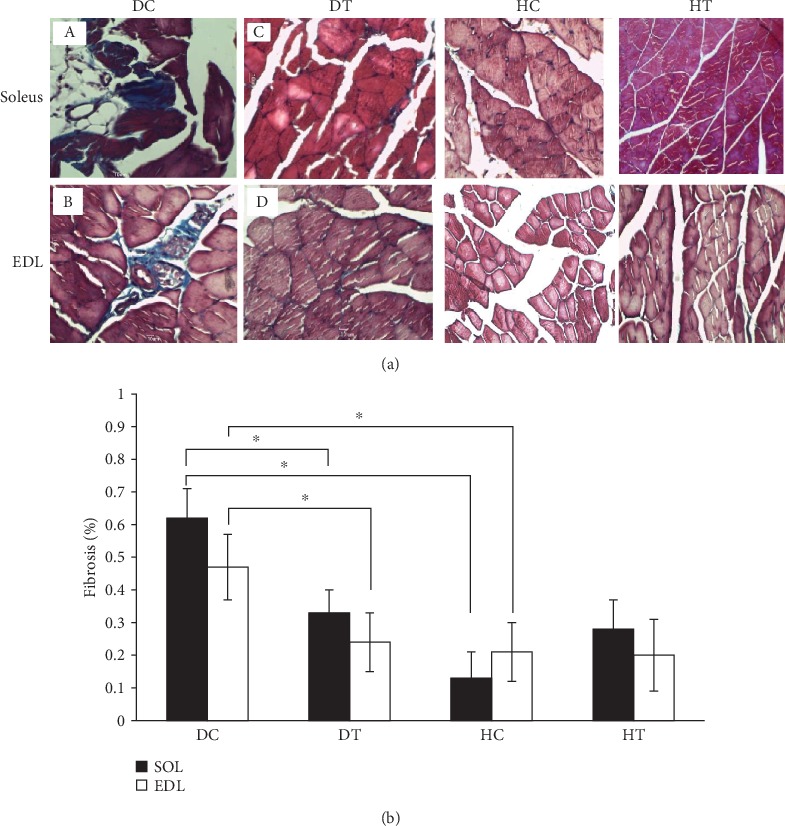
Muscle fibrosis in soleus and EDL muscles in different groups. Masson's trichrome staining was used to determine the amount of collagen in the muscle tissue. (a) (A) The soleus muscle and (B) the EDL muscle of the DC group. (C) The soleus muscle and (D) the EDL muscle of the DT group. Muscle fibrosis is in blue. (b) Among the soleus muscles, the highest level of collagen was in the soleus muscle of the DC group (A) (*P* ≤ 0.05). The highest level of collagen among EDL muscles was in the DC group (B) (*P* ≤ 0.05). The exercise training significantly decreased the amount of tissue fibrosis in the DT group in both muscles (C and D) (*P* ≤ 0.05). Scale bar on pictures is 10 *μ*m.

Collagen levels in soleus muscles and EDL in the DC group were higher than those in the HC group indicating the occurrence of muscle fibrosis in diabetic animals (*P* = 0.002 and *P* = 0.04, respectively). Soleus muscle collagen levels in the DC group showed a higher increase than those in other groups (Figure 2) and the increase between DC and HC groups was significant (*P* = 0.02).

EDL Muscle collagen level in DC Group showed a higher increase than other groups (Figure 2) and the increase was significant between the DC and HC groups (*P* = 0.048). On the other hand, six weeks of endurance exercise led to a significant reduction in collagen levels between the DT and DC groups in soleus and EDL muscle (*P* = 0.05 and *P* = 0.001, respectively) indicating that the endurance training was associated with a decrease in muscle fibrosis (Figure 2).

### 3.3. Changes in Protein Levels of NRG1 and ErbB2

**Figure 3:**
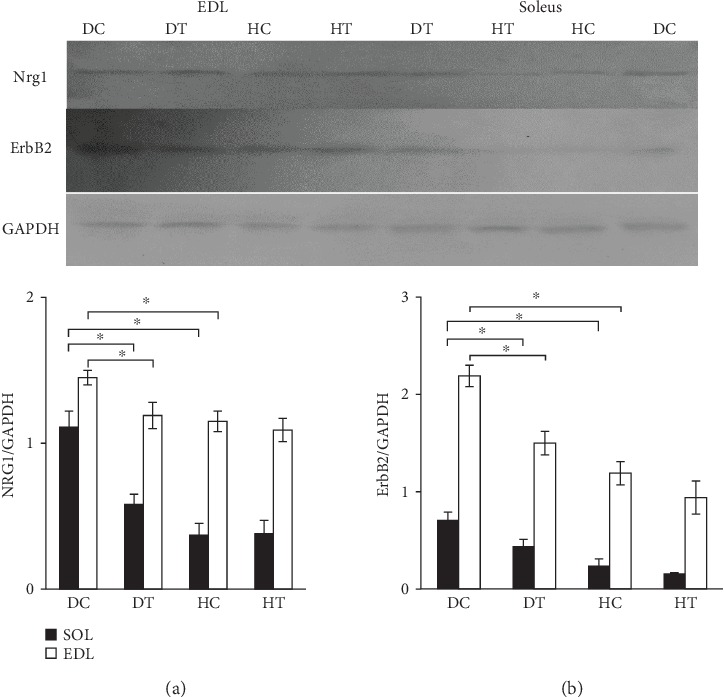
Expression of NRG1 (a) and ErbB2 (b) is demonstrated by Western blotting normalized to GAPDH. Panels above the graph are representative Western blot images from different groups. ^∗^*P* < 0.05 vs. control rats (two-way ANOVA). DC: diabetic control rats; DT: diabetic and trained; HC: health and control; HT: health and trained. Sol: soleus; EDL: extensor digitorum longus. Five rats in each group were utilized for this analysis. The analysis was performed in triplicate.

Diabetes mellitus increased the expression of protein levels of NRG1 and ErbB2 in skeletal muscles of STZ diabetic rats. NRG1 protein content in the DC group significantly increased in the soleus muscle than that in the other groups (Figure 3), and the increase was significant between the DC and HC groups (*P* = 0.006). NRG1 protein content in the DC group in the EDL muscle increased more than that in the other groups, and the increase was significant between the DC and HC groups (*P* = 0.03). On the other hand, six weeks of exercise training could decrease the expression of NRG1 and ErbB2 in skeletal muscles of STZ diabetic rats compared to the DC group rats. NRG1 protein content in the DT group was significantly lower than that in the DC group in the soleus and EDL muscles (*P* = 0.335 and *P* = 0.04, respectively).

ErbB2 protein content in the DC group in the soleus muscle was higher than that in the other groups (Figure 3), and the increase between the DC and HC groups was significant (*P* = 0.033). ErbB2 protein content in the DC group in EDL muscle was higher than that in the other groups, and the increase was significant between the DC and HC groups (*P* = 0.001). On the other hand, six weeks of exercise training could decrease the expression of ErbB2 in skeletal muscles of STZ diabetic rats compared to the DC group rats. ErbB2 protein content significantly decreased in the DT Group compared to the DC group in soleus and EDL muscles. (*P* = 0.33% and *P* = 0.001, respectively).

## 4. Discussion

The present research indicated that diabetes increased NRG1 and ErbB2 protein levels and rates of skeletal muscle fibrosis in STZ diabetic rats. On the other hand, six weeks of endurance exercise training decreased the NRG1 and ErbB2 protein content and skeletal muscle fibrosis of STZ diabetic rats. This effect was associated with a reduction in blood glucose levels in trained diabetic rats. The results suggested that the exercise training against diabetes-induced hyperglycemia could be associated with an increase in insulin levels, a decrease in blood glucose, and adjustment of NRG1 and ErbB2 proteins.

We initially assumed that diabetes might interrupt the expression level of NRG1 and ErbB2 proteins. In skeletal muscles, diabetes caused a glomerular hemostasis disorder in muscle cells, and on the other hand, NRG1 could cause the glomerular hemostasis in skeletal muscles [24]. To assess the hypothesis, rats had diabetes for 8 weeks (2 weeks before training and 6 weeks after training). This period of being diabetic was associated with the weight loss and increased blood glucose levels compared to the HC group. NRG1 and ErbB2 protein levels significantly increased in both muscles of the diabetic group than the healthy one; and the results confirmed our first hypothesis. Increased proteins have been also reported in many other illnesses such as lung cancer [25], cardiomyopathy [26], Charcot–Marie–Tooth disease (CMT) [27]. The excessive concentration increase in NRG can be dangerous to cells. Too much increase in NRG or ErbB2 receptors in different cells can be detected by the nonregulation of cell growth control and cancer pathologies [28]. This finding indicated a subtle relationship of expression/activation regulation of available ErbB and NRG receptors. Some studies have considered the increased expression of NRG1 in the surreal nerve of diabetic rats as a factor in increasing the activity of ErbB2 and contribution to the development of neuropathic pain [10]. In addition, McGuire et al. reported that the continuous activation of ErbB2 contributed to the development of peripheral neuropathy in diabetes [29]. On the other hand, some studies indicated that NRG1 weakened the oxidative stress in microglial and heart cells [30, 31]. It is well accepted that the increased production of mitochondrial ROS is associated with insulin resistance and diabetes and on the other hand, the excessive increase in production of ROS can interfere in calcium hemostasis that leads to defect in the contraction [32]. Therefore, the role of NRG1 in reducing the oxidative stress associated with diabetes may be a reason for increasing NRG1 in diabetic rats. Given the above cases, the increase in NRG1 and ErbB2 should have a protective and controlling role in improving conditions of diabetes.

In the present study, the endurance exercise in diabetic rats could decrease NRG1 and ErbB2 levels in soleus and EDL muscles. We assumed that the endurance exercise might alter the expression of NRG1 and ErbB2 proteins in diabetic rats. After six weeks of endurance sports, NRG1 and ErbB2 levels decreased in diabetic rats. The reason why the exercise training decreased NRG1 and ErbB2 levels in soleus and EDL muscles may be due to the fact that it could significantly decrease blood glucose levels in the diabetes mellitus group. In skeletal muscles, the glucose disposal could be also activated by muscle contraction activity. The ability of muscle contractions to stimulate glucose disposal can be responsible for some beneficial effects of exercise in diabetes through a PI3K-dependent pathway. The activation of PI3K plays a central role in the transfer of GLUT-4 vesicle and increased glucose transfer [33]. AMPK is a major regulator of glucose uptake due to the exercise. Muscle contraction is activated through liberation of Ca^+2^ and also activates CaMKs. AMPK and CaMKs regulate the glucose uptake from exercise independent of insulin. This process is done by stimulating the transfer of bags containing GLUT4 to the cell membrane and its combination with glucose [34]. Signaling pathway of NRG1 is not clear in contraction that induces the glucose uptake. Despite the fact that caMKII is not directly activated by NRG1, this kinase is partly interacting with the signaling pathway of NRG1 in a way that the prevention of this kinase impairs effects of NRG1 on the glucose uptake [6]. Since many biological processes can be regulated through ErbB, it is difficult to predict functional consequences of increased ErbB and its phosphorylation in diabetes [24].

Histologic results of the present study indicated that diabetes increased the muscle fibrosis in muscles of diabetic rats. Fibrosis is considered a sign of muscular dystrophy [35]. It is due to the accumulation of extracellular matrix and sometimes the quality change of ECM. A reason for fibrosis is the pathologic response to muscle weakening due to the increased blood glucose leading to the structural and functional disorders in the muscle [36]. Changes in ECM and thickness of the basement membrane are considered structural markers in all target organs of diabetes [37]. The empirical data supports the effective hyperglycemic roles and biochemical pathways in ECM regeneration changes. Hyperglycemia can play a role in the ECM regeneration through metabolic and hemodynamic pathways [14]. In the present study, an increase in the fibrosis in soleus and EDL muscles might be caused by increased blood glucose levels due to diabetes. On the other hand, the endurance training was associated with a decrease in the amount of fibrosis by decreasing the blood glucose in the diabetic group. Previous studies evaluated structural changes in the skeletal muscle fibers of diabetic animals and the weakness of fibers due to chronic diabetes [38] as well as structural changes in skeletal muscles of rats that became diabetics by streptozotocin [39]. Certainly, physiological functions of skeletal muscles were impaired during the diabetes stage, and soleus and EDL muscles, which were investigated in the research, showed fibrotic changes. The diabetic model due to the prescribed STZ in the present study also proved that certain changes could be traced in the myofibers such as the tissue degeneration (fibrosis) that was a definite type of myopathy. On the other hand, as shown in images of tissue sections of diabetic specimens, collagen accumulation in myofibers might indicate that in diabetes, which is usually associated with microangiopathic events, in addition to ischemia as an important factor in the degeneration of myofibers [40], muscle fibers following the diabetic myopathy are much weaker than normal specimens and have decreased numbers [41]. On the other hand, increasing the amount of tissue fibrosis is associated with increasing growth factors such as epithelial growth factor (EGF) and NRG1 as a member of this family [42]. Due to higher levels of fibrosis in diabetic groups, the increased NRG1 and ErbB2 in the present study may be attributed to increased fibrosis. Given the role of endurance activity in reducing the creation of fibrosis in diabetes and the association of fibrosis with growth factors, it can be argued that exercise training prevents increased amounts of NRG1 and ErbB2 proteins in soleus and EDL muscles of diabetic group rats by inhibiting or reducing the amount of fibrosis.

## 5. Conclusion

In summary, results of the present study strongly suggested a mechanism under which the diabetes mellitus increased the fibrosis and NRG1/ErbB2 in skeletal muscles of STZ diabetic rats. On the other hand, endurance exercise training decreased blood glucose levels, muscle fibrosis, and NRG1/ErbB2 activities in diabetic skeletal muscles. More studies should be conducted on the role of fibrosis, increased NRG1/ErbB2, and effect of endurance exercise training on them. Therefore, it is suggested utilizing exercise as a nonpharmacological intervention to reduce complications of diabetes.

## Figures and Tables

**Table 1:** Changes of body mass after streptozotocin (STZ) injection (pretraining) and after 6-week exercise treatment (posttraining). Data is shown as the mean ± SEM. DC: diabetic control rats; DT: diabetic and trained; HC: health and control; HT: health and trained.

	DC	DT	HC	HT
Before training	234.00 ± 15.05	231.50 ± 9.73	233.50 ± 10.28	234.00 ± 8.75
After training	198.37 ± 10.72^∗^	217.88 ± 18.90	258.50 ± 8.33	247.77 ± 9.39

Data is shown as the mean ± SEM. ^∗^Significant at *P* < 0.05.

## Data Availability

The history, protein analysis, and all other relevant data used to support the findings of this study are included within the article.
